# Women's views and experiences of breastfeeding during the coronavirus disease 2019 pandemic: A systematic review of qualitative evidence

**DOI:** 10.1111/mcn.13708

**Published:** 2024-08-09

**Authors:** Padma Uma Devi, Sarah Beake, Yan‐Shing Chang

**Affiliations:** ^1^ Florence Nightingale Faculty of Nursing Midwifery and Palliative Care, King's College London London UK

**Keywords:** breastfeeding, breastfeeding support, coronavirus disease 2019, meta‐aggregation, qualitative, systematic review

## Abstract

The coronavirus disease 2019 pandemic affected breastfeeding women in various ways. Understanding their experiences during the pandemic is crucial for informing actionable recommendations, evidence‐based strategies and future policies to support breastfeeding during global pandemics. This review aimed to synthesise qualitative evidence on women's breastfeeding perceptions, experiences and support needs during the pandemic. The Joanna Briggs Institute's (JBI) guidelines on systematic reviews of qualitative evidence were followed. MEDLINE, Embase, CINAHL and Web of Science Core Collection databases were searched. Methodological quality of included papers was assessed using JBI's checklist for qualitative research. The synthesised findings were generated using JBI's meta‐aggregation approach. The JBI ConQual process was used to rank each synthesised finding. Fifty‐two papers were included. The synthesised findings included: (1) women's awareness and commitment to breastfeeding during the pandemic, (2) the multifaceted breastfeeding experiences of women during the pandemic, (3) breastfeeding practices and challenges for working women, (4) professional support during the pandemic: navigating breastfeeding in an evolving health care context and (5) family and peer support groups during the challenging times of the pandemic. Breastfeeding women require clear information, accessible in‐person lactation support, family emotional support, food security and protection of psychological well‐being. The review reported diverse breastfeeding experiences, from social support challenges to positive aspects like remote work. Breastfeeding support and lactation consultants should be considered as essential services in future pandemics. Food security is crucial for breastfeeding households. Lactation services could prioritise face‐to‐face consultations for physical challenges and providing online informational support. Future research could explore innovative breastfeeding education strategies.

## Key messages

- Breastfeeding women, during the pandemic, faced challenges due to disrupted post‐natal care, cancelled breastfeeding classes and insufficient health care professional support, leading to feelings of neglect, isolation and uncertainty.
- During the pandemic, breastfeeding women received limited face‐to‐face support from family and friends. In the absence of in‐person support, online platforms emerged as valuable resources for breastfeeding guidance.
- In future pandemics, breastfeeding support and designate lactation consultants should be prioritised as essential services to ensure uninterrupted breastfeeding support.
- Innovative strategies, including online and real‐time breastfeeding education and support, should be further developed and rigorously evaluated to effectively address breastfeeding challenges during pandemics.

## Graphical abstract

Breastfeeding women require clear information, accessible in‐person lactation support, family emotional support, food security and protection of psychological well‐being during a pandemic. The review reported diverse breastfeeding experiences, from social support challenges to positive aspects like remote work. Food security has been found to be crucial for breastfeeding households.

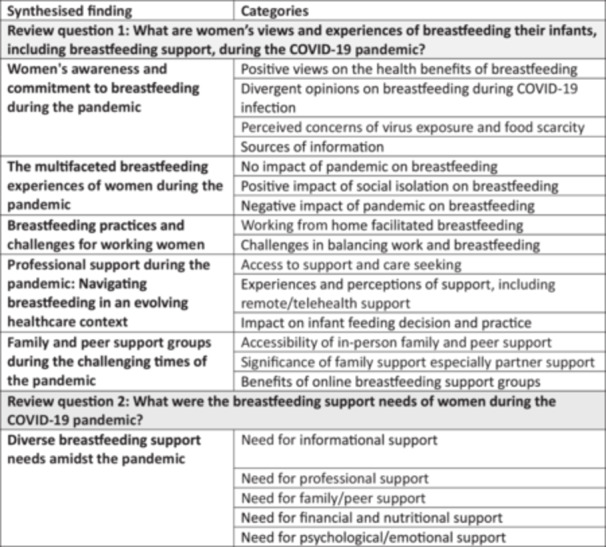

## 1 INTRODUCTION

The World Health Organisation (WHO) recommends exclusive breastfeeding for the first 6 months of an infant's life (World Health Organization, 2024), recognising the numerous benefits for both infants and mothers (Victora et al., 2016). However, the onset of the highly contagious coronavirus disease 2019 (COVID‐19) pandemic prompted the implementation of a series of preventive measures, including lockdowns and social distancing (Girum et al., 2021). These measures affected maternal and infant care, including breastfeeding (Sakalidis et al., 2022). On the one hand, these measures resulted in limited access to both professional and informal support networks, including family, friends and peer groups (Coxon et al., 2020; Snyder & Worlton, 2021) and also adoption of telemedicine (Omboni et al., 2022). On the other hand, they brought about positive effects such as the option to work from home and a reduction in visitors (Oggero & Wardell, 2022).

Despite the WHO's recommendation for skin‐to‐skin contact and direct breastfeeding with hygiene precautions for mothers with suspected or confirmed COVID‐19 infection (World Health Organization, 2020), concerns about virus transmission prompted many hospitals to separate mother–infant pairs (Gonçalves‐Ferri et al., 2021; Liao et al., 2020), thereby affecting both breastfeeding initiation and continuation (Bernstein et al., 2023).

Previous reviews on this topic adopted a narrative approach with focus on mental health and breastfeeding (Pacheco et al., 2021), primarily focused on high‐income countries (Adesanya et al., 2022) or restricted to breastfeeding outcomes after COVID‐19 social containment measures with unclear maximum infant age (Antoniou et al., 2023). Consequently, a significant gap in the literature remains for a comprehensive global qualitative systematic review exploring women's breastfeeding experiences and their support needs during the COVID‐19 pandemic. Understanding these experiences and needs from the perspectives of women amid the challenges and opportunities presented by the pandemic is essential for informing actionable recommendations, evidence‐based strategies and policy development to ensure sustained breastfeeding support during future pandemics globally.

## 2 METHODS

The Joanna Briggs Institute's (JBI) systematic reviews of qualitative evidence guidelines were followed (Lockwood et al., 2020). The decision to adopt a qualitative approach was driven by its capacity to explore participants' experiences and viewpoints (Evans, 2002). The protocol for this review was registered on PROSPERO (CRD42023417952).

The review questions were:

1. What are women's views and experiences of breastfeeding their infants, including breastfeeding support, during the COVID‐19 pandemic?
2. What were the breastfeeding support needs of women during the COVID‐19 pandemic?

### 2.1 Inclusion/Exclusion criteria

#### 2.1.1 Participants

Studies were considered if they involved post‐natal women of all parties who provided breast milk to their infants (0–24 months) during the COVID‐19 pandemic. Studies focusing on women with infants having medical complications, pregnant women's perspectives on breastfeeding, and the perspectives and experiences of health care professionals in providing breastfeeding support during the pandemic were excluded. Studies reporting post‐natal women's views and experiences of breastfeeding support, including breastfeeding education received during their pregnancy, were included.

#### 2.1.2 Phenomenon of interest and context

This review considered studies that explored post‐natal women's views and experiences concerning breastfeeding, including breastfeeding support during the COVID‐19 pandemic, irrespective of the geographical location or setting. Studies focusing on women's post‐partum experiences were considered only if they discussed breastfeeding experiences, views, support or education received.

#### 2.1.3 Types of studies

This review included qualitative studies, qualitative data from mixed methods research and responses from open‐ended survey questions published in peer‐reviewed journals. Quantitative studies, grey literature, conference abstracts, dissertations, guidelines, opinion papers, letters to editors, commentaries, policy documents and reviews were excluded. Only primary studies published in English from January 2020 were considered since the initial case of COVID‐19 was reported in December 2019 (World Health Organization, 2021).

### 2.2 Search strategy

Following the JBI guidelines, a three‐step process was undertaken (Lockwood et al., 2020). The review centred on two key concepts: breastfeeding and COVID‐19. In the initial preliminary search phase, a focused search was conducted in MEDLINE and CINAHL to identify relevant free‐text words within titles and abstracts and index terms linked to breastfeeding. A COVID‐19 search hedge was used in the CINAHL database (Campbell, 2023) and adapted for the Web of Science. For MEDLINE and Embase, the COVID‐19 limit option provided by these databases was used. In the second phase, which was the final search conducted on 17 March 2023, tailored searches were executed in MEDLINE (Ovid), Embase (Ovid), CINAHL (EbscoHost) and Web of Science Core Collection. Lastly, the reference lists of included articles were manually examined to identify any other relevant studies. The search strategy for all databases is displayed in supplementary material 1.

### 2.3 Study selection and assessment of the methodological quality

The search results from the databases were imported into Covidence for screening. PUD conducted all the title and abstract screening, with 10% randomly and independently screened by YSC. Full texts were independently screened by two reviewers (PUD/YSC and PUD/SB) based on eligibility criteria, with any differences resolved through discussion. Critical appraisal was independently conducted by two reviewers (PUD/YSC and PUD/SB) using the JBI checklist for qualitative research (Lockwood et al., 2020). Any discrepancies were resolved through discussion.

To assess the confidence of evidence for each synthesised finding, the JBI ConQual process was followed (Lockwood et al, 2020). Each paper included in a synthesised finding was initially ranked from ‘high' for qualitative papers to ‘low’ for text and opinion papers. A dependability score was then applied, to assess the appropriateness of each paper, based on its critical appraisal scores. This was followed by assigning a level of credibility by checking if findings included were supported. A final score is then allocated to each synthesised finding (Table 1).

**Table 1:** ConQual summary of findings.

Synthesised findings	Type of research	Dependability	Credibility	ConQual score	Comments
Review question 1: What are women's views and experiences of breastfeeding their infants, including breastfeeding support, during the COVID‐19 pandemic?					
1.1 Women's awareness and commitment to breastfeeding during the pandemic	Qualitative—High	Downgrade one level—Moderate*	Remains unchanged**	Moderate	Findings included from 15 papers. *Downgraded one level as the majority of papers (7 out of 15) scored 2–3 out of 5 for questions relating to appropriateness of conduct of the research **Remains unchanged as all findings unequivocal
1.2 The multifaceted breastfeeding experiences of women during the pandemic	Qualitative—High	Downgrade one level—Moderate*	Remains unchanged**	Moderate	Findings included from 27 papers. *Downgraded one level as the majority of papers (17 out of 27) scored 2–3 out of 5 for questions relating to appropriateness of conduct of the research **Remains unchanged as all findings unequivocal
1.3 Breastfeeding practices and challenges for working women	Qualitative—High	Downgrade one level—Moderate*	Remains unchanged**	Moderate	Findings included from 11 papers. *Downgraded one level as the majority of papers (9 out of 11) scored 2–3 out of 5 for questions relating to appropriateness of conduct of the research **Remains unchanged as all findings unequivocal
1.4 Professional support during the pandemic: Navigating breastfeeding in an evolving health care context	Qualitative—High	Downgrade one level—Moderate*	Remains unchanged**	Moderate	Findings included from 30 papers. *Downgraded one level as the majority of papers (20 out of 30) scored 2–3 out of 5 for questions relating to appropriateness of conduct of the research **Remains unchanged as all findings unequivocal
1.5 Family and peer support groups during the challenging times of the pandemic	Qualitative—High	Downgrade one level—Moderate*	Remains unchanged**	Moderate	Findings included from 20 papers. *Downgraded one level as the majority of papers (13 out of 20) scored 2–3 out of 5 for questions relating to appropriateness of conduct of the research **Remains unchanged as all findings unequivocal
Review question 2: What were the breastfeeding support needs of women during the COVID‐19 pandemic?					
2. Diverse breastfeeding support needs amidst the pandemic	Qualitative—High	Downgrade one level—Moderate*	Remains unchanged**	Moderate	Findings included from 30 papers. *Downgraded one level as the majority of papers (19 out of 30) scored 2–3 out of 5 for questions relating to appropriateness of conduct of the research **Remains unchanged as all findings unequivocal

### 2.4 Data extraction and synthesis

Characteristics of each included paper such as author name, country, participant characteristics, methodology, data collection and analysis methods and key findings were extracted in a tabular format. Meta‐aggregation, JBI's three‐stage process for synthesising data was used to synthesise the findings (Lockwood et al., 2020). Findings, including author observations or themes (illustrated with participant quotes where available), were extracted from the results section of the included papers. A Microsoft excel sheet was used for the purpose of extracting the findings. In the subsequent stage, categories were constructed by aggregating findings with similar meanings. Findings without supporting quotations (unsupported findings) were excluded from category formation. Ultimately, the synthesised findings were derived by combining two or more similar categories (Lockwood et al., 2020). PUD conducted extraction of findings and analysis and verified by YSC and SB. All the authors agreed on the final synthesised findings.

### 2.5 Ethics statement

This is a systematic review and therefore ethical approval is not required.

## 3 FINDINGS

### 3.1 Characteristics of included studies

**Figure 1:**
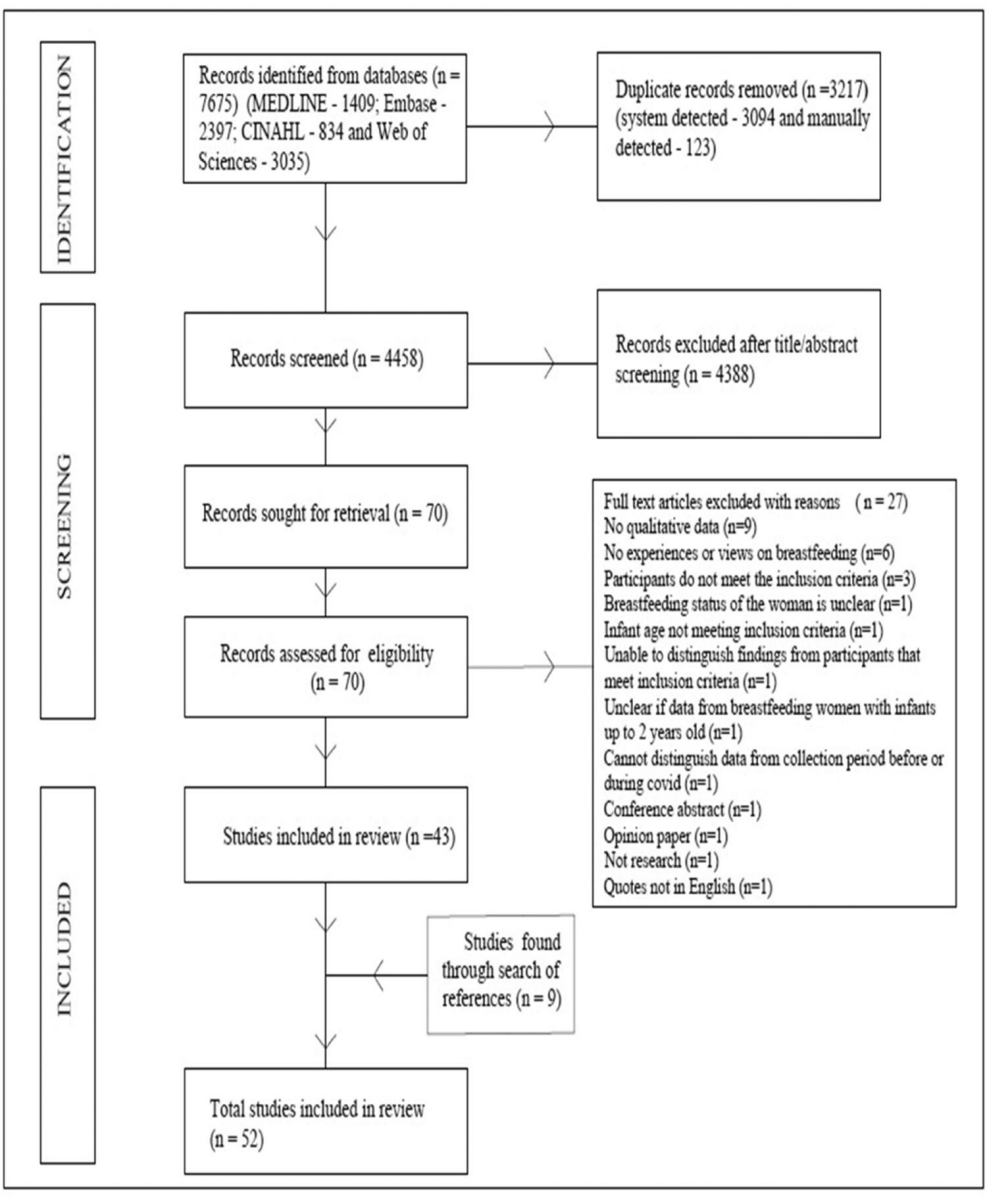
Flowchart of selection process (adapted from PRISMA 2020 flow diagram) (Page et al., 2021).

A total of 7675 papers were identified through database searches. After removing duplicates and title and abstract screening, 70 full texts were retrieved and screened based on predetermined eligibility criteria among them, 27 papers were excluded (Figure 1). Nine papers were identified from the reference lists of the 43 included papers. Overall, 52 papers were included.

Thirty‐nine of the included papers were from high‐income countries: 13 from the United States, six from the United Kingdom and Canada, five from Australia, three from Norway, two from Ireland and New Zealand, and one each from Italy, Denmark, Spain, Belgium, Netherlands, Switzerland, Hong Kong (China), Malta, Saudi Arabia and Israel. Among these, four were multinational studies (Atchan et al., 2023; Ceulemans et al., 2021; Sweet, Muller, et al., 2022; Turner et al., 2023). Six papers were from upper‐middle‐income countries: two from Indonesia and Thailand, one from South Africa and Turkey. There were six studies from lower–middle–income countries: three from Kenya, two from India and one from Lebanon. There was only one study from a low‐income country, i.e., Uganda (Atuhaire et al., 2021). Twenty‐nine studies were qualitative and utilised interviews or focus groups. The remaining were mixed methods studies or cross‐sectional surveys with open‐ended questions. Characteristics of the included studies are shown in Table 2.

**Table 2:** Study characteristics of included papers.

Author, year and country	Aim of study	Participants	Methodology	Method (relevant to the review)	Data analysis (relevant to the review)	Key findings (relevant to the review)
Agrina et al. (2022) (Indonesia)	To examine breastfeeding practices and identify factors supporting exclusive breastfeeding during the COVID‐19 pandemic.	Mothers with infants >6 months to 24 months (156 for quantitative and 12 for qualitative study)	Mixed methods (sequential explanatory design)	Semi‐structured in‐depth interviews	Thematic data analysis	Participants considered husbands and family members as significant sources of support. The assistance from health care workers was deemed insufficient.
Ahoya et al. (2023) (Kenya)	To understand COVID‐19 pandemic's effect on health service delivery, food systems, maternal and infant nutrition practices, including breastfeeding practices.	Pregnant women (*n* = 16), breastfeeding women with an infant aged 0–23 months (n = 31 including 15 COVID‐19 infected cases), health workers (*n* = 10), community health volunteers (*n* = 10), food vendors and stakeholders (government and implementing partners)	Implementation research study	In‐person, in‐depth interviews with breastfeeding women	Transcripts were coded, and dominant themes were presented	Themes: “(1) disruptions in early initiation of breastfeeding, (2) disruptions in exclusive breastfeeding, (3) skipping meals, and (4) reducing quantity and/or diversity of foods eaten” (p.7‐9)
Aşcı et al. 2022 (Turkey)	To explore breastfeeding experiences of women diagnosed with COVID‐19 while breastfeeding.	14 women diagnosed with COVID‐19 while breastfeeding	Qualitative study	One‐to‐one telephonic semi‐structured interviews	Thematic analysis	Three main themes: “(1) increased emotional load, (2) breastfeeding during the disease, (3) perceived social support and needs” (p.1) Many women preferred to continue breastfeeding during the illness despite difficulties ensuring breast milk benefits for their child. Professional support was generally considered inadequate.
Atchan et al. (2023) (Australia and New Zealand)	To investigate early parenting and infant feeding experiences of new mothers during the COVID‐19 pandemic.	Mothers (*n* = 27) who gave birth during the pandemic	Interpretive qualitative approach	In‐depth, semi‐structured interviews	Thematic analysis	Four main themes: “(1) feeding decisions and practices, (2) the COVID‐19 breastfeeding experience, (3) experiences of support, (4) psychological impacts” (p.4) Many women found the peaceful environment due to social distancing beneficial. For some, the restriction due to social distancing created distress and negatively impacted them because it limited their access to professional assistance and social support to establish breastfeeding or deal with feeding challenges.
Atuhaire et al. (2021) (Uganda)	To explore the lived experiences of women who had recently recovered from post‐partum depression.	30 mothers (10–18 weeks post‐partum) who had recovered from post‐partum depression	Phenomenological study	In‐depth semi‐structured interviews	Thematic analysis	Some mothers experienced low or absence of breast milk production due to reduced appetite subsequent to stress/anger.
Badr and Alghamdi (2022) (Saudi Arabia)	To investigate breastfeeding experiences of women during the COVID‐19 pandemic.	18 breastfeeding women	Descriptive phenomenological qualitative study	Semi‐structured interviews by telephone	Thematic analysis	Four main themes: “(1) the breastfeeding experience (positive and negative), (2) support and resources that helped mothers continue breastfeeding during COVID‐19, (3) facilitators of breastfeeding, (4) challenges preventing mothers from continuing to breastfeed” (p.4) Husbands, mothers, friends, lactation consultants and social media supported breastfeeding. Distance working acted as a facilitator. Long working hours (in health care settings) and short breaks were possible challenges.
Brown and Shenker (2021) (United Kingdom)	To investigate mothers' breastfeeding practices, experiences and support during the COVID‐19 pandemic.	1219 mothers who breastfed their infants (0–12 months) during the pandemic	Mixed methods online survey study	Questionnaire with closed and open questions	Thematic analysis	Three broad categories: (1) positive breastfeeding experience (2) negative experience (3) no effect. Positive impact sub‐themes: 'more time to focus, fewer visitors, more privacy, increased responsive feeding, greater partner support, and a delay of return to work outside the home' (p.8)Negative impact sub‐themes: 'a lack of face‐to‐face support, a lack of social support, stress of caring for other children, intense focus on breastfeeding, no experience of breastfeeding in public, and work concerns' (p.9)
Cassar and Spiteri (2022) (Malta)	To explore the experiences regarding antenatal education received by first‐time mothers during the COVID‐19 pandemic.	Nine women with term infants who attended antenatal classes during the pandemic (virtual or face‐to‐face)	Qualitative study	Semi‐structured virtual interviews	Thematic analysis	Mothers viewed antenatal education useful regarding the theoretical aspects of infant feeding. Few mothers felt these classes pressured them to breastfeed.
Ceulemans et al. (2021) (Belgium, Norway, Netherlands, Switzerland, Ireland and United Kingdom)	To explore COVID‐19 pandemic's impact on pregnant and breastfeeding women's beliefs regarding coronavirus, COVID‐19 vaccine willingness, perinatal experiences and breastfeeding practices.	Pregnant women (*n* = 6661) and breastfeeding women (*n* = 9402)	Cross‐sectional study	Web‐based survey	Not stated	Many women breastfed for longer since they were working from home, had no visitors and wanted to provide protection (antibodies) through breast milk. Some women feared infecting the child during breastfeeding, desired the need to attend breastfeeding support groups and had feelings of not having time for breastfeeding.
Cohen and Botz (2022) (United States)	To explore the experiences of parents and lactation professionals on feeding human milk to infants during the COVID‐19 pandemic.	24 parents who fed their babies human milk and 13 lactation specialists	Qualitative study with some elements of visual ethnography	In‐depth unstructured interviews via videoconferencing	Content analysis	Four themes: '(1) the loneliness of lactation during the pandemic, (2) the construction of human milk as a resource to cope with the crisis, (3) the (in)visibility of lactation amidst heightened multitasking, (4) the sense of connection created by human milk feeding at a time of unprecedented solitude' (p.1)
DeYoreo et al. (2023) (United States)	To investigate the impact of the COVID‐19 pandemic on breastfeeding support and experiences.	1617 parents who gave birth in one of the following 'birth cohorts': August–December 19 (*n* = 519), March–May 20 (*n* = 522), or June–August 21 (*n* = 576)	Cross‐sectional study	Survey with an open‐ended question	Responses were manually coded (inductive approach) into thematic categories	Positive impact – some parents breastfed longer to give antibodies to the infant or due to working from home. Negative impact – disruptions in professional and social support, reduced breast milk supply due to stress.
DeYoung and Mangum (2021) (United States)	To investigate the COVID‐19 pandemic's impact on pregnancy, childbirth and postpartum, including infant feeding experiences.	192 pregnant and postpartum women	Cross‐sectional study	Web‐based survey with open‐ended questions	Coding was done, and additional themes were identified	Difficulty accessing lactation and social support led to early breastfeeding cessation for some participants. Working from home allowed some respondents to maintain breastfeeding without pumping.
Eri et al. (2022) (Norway)	To investigate women's experiences through pregnancy, childbirth and the early postpartum period amid the COVID‐19 pandemic.	806 women who had given birth between Mar and Dec 20 in Norway and had answered at least one open‐ended question	Mixed‐methods survey	Web‐based questionnaire with open‐ended questions	Inductive thematic analysis	Participants felt that breastfeeding assistance was limited, and the consultations were brief and lacked empathy.
Fry et al. (2021) (Canada)	To investigate changes to feeding practices perceived by caregivers of infants (<6 months) during the COVID‐19 pandemic.	335 primary caregivers of infants < 6 months	Cross‐sectional study	Online questionnaire with open and closed‐ended questions	Data were analysed thematically	Positive experiences included more time to focus on breastfeeding and comfort nursing. Lack of access to lactation support and resolving breastfeeding problems over the phone was challenging.
Fumagalli et al. (2022) (Italy)	To investigate the experiences of COVID‐19‐infected women who delivered during the pandemic.	22 COVID‐19‐positive women who delivered during March–April 20	Qualitative study (interpretative phenomenological approach)	Semi‐structured interviews via telephone/video call (*n* = 21) or face‐to‐face (*n* = 1)	Thematic data analysis	COVID‐19‐positive women were worried about prolonging their contact with their newborns during breastfeeding due to concerns for disease transmission, despite adhering to preventive measures.
Glassman et al. (2022) (United States)	To examine breastfeeding preparedness and experiences of mothers when the breastfeeding support services are provided by a paediatrician/International Board‐Certified Lactation Consultant (MD/IBCLC).	28 mothers who had an MD/IBCLC visit	Mixed methods	Semi‐structured telephone interviews	Framework method	The video visits during the pandemic allowed breastfeeding from one's usual setting and avoiding potential exposures. Even though the isolation reduced social help, it allowed more time to focus on breastfeeding, thereby passing immunity to infants.
Goyal et al. (2022) (United States)	To investigate experiences of women in the initial 6 weeks postpartum and assess postpartum depression risk during the COVID‐19 pandemic.	262 women who gave birth to live infants	Convergent, parallel mixed methods study	Questionnaire with open‐ended questions	Content analysis	Some mothers felt breastfeeding was challenging due to reduced access to lactation consultants and social support.
Hill et al. (2023) (United States)	To examine the return to work experiences of nurses working in emergency departments.	Nurses who returned from parental leave within six months (one‐on‐one interview, *n* = 11) or within the previous two years (focus groups, *n* = 8 in three focus groups)	Qualitative descriptive study	One‐on‐one interviews (in‐person, phone, Zoom) Focus groups (in‐person, Zoom)	Thematic analysis	Participant's ability to take appropriate lactation breaks depended on their department's staffing and patient load during COVID‐19.
Jackson et al. (2021) (United Kingdom)	To examine the psychological experiences of women during the postpartum period at different time points of the COVID‐19 pandemic.	24 women (12 women at each time point) who delivered live infants during 3 months before the interview	Qualitative study	Semi‐structured interviews (via telephone or Zoom)Timepoint 1 and 2 (about 1 month following the introduction and lifting of the social distancing restrictions, respectively)	Thematic analysis	Some participants perceived the visitor restriction as a supportive factor in establishing breastfeeding and facilitating responsive feeding. Women with breastfeeding difficulties perceived the reduced face‐to‐face visits and lack of breastfeeding support (health care professionals or peers) as challenging.
Jacob et al. (2022) (United States)	To examine mothers' views and experiences of delivery and postpartum care received during the pandemic.	13 postpartum mothers	Phenomenological study	Unstructured interviews via Microsoft Teams	Thematic analysis	Adverse breastfeeding experiences were reported due to a perceived lack of support from lactation consultants.
Jensen et al. (2022) (Denmark)	To investigate the experiences, health literacy and risk perception of women with recent gestational diabetes mellitus during COVID‐19 pandemic.	Women with recent gestational diabetes mellitus (*n* = 11) (infant age 2–11 months)	Qualitative study	Semi‐structured interviews (using telephone or Skype)	Qualitative content analysis	Mothers were worried about managing potential breastfeeding difficulties if they contracted COVID‐19.
Jiravisitkul et al. (2022) (Thailand)	To determine the facilitators and barriers to breastfeeding continuation on return to work following maternity leave in the hospital workplace.	65 female permanent employees (seven employees participated in the focus group discussion)	Mixed methods study	Focus group discussion (using Google Meet) included subgroups of four and three participants with successful and unsuccessful breastfeeding continuation following return to the workplace	Thematic coding analysis	Women were worried about pumping milk in the hospital due to concerns about hygiene during the pandemic. The increased workload among health workers during the pandemic created stress and affected the breast milk supply.
Joy et al. (2020) (Canada)	To explore parents' postpartum experiences during the COVID‐19 pandemic.	68 mothers with infants aged 0–12 months	Qualitative study (feminist poststructuralism)	Open‐ended online survey	Discourse analysis	Due to the isolation of COVID‐19, mothers experienced reduced worries about breastfeeding in front of others.
Kolker et al. (2021) (Canada)	To explore the lived experiences of pregnant and postpartum women during the COVID‐19 pandemic.	12 postpartum women	Qualitative descriptive approach	Open‐ended semi‐structured interviews by phone	Descriptive thematic analysis	Women described limited access to breastfeeding classes or support during the pandemic, thus leaving them with many unanswered questions regarding breastfeeding. Some women found online breastfeeding classes challenging and preferred in‐person support.
Magnazi et al. (2022) (Israel)	To examine COVID‐19 pandemic's impact on the breastfeeding practices of women with infants aged below 6.5 months.	580 mothers who breastfed their infants (<6.5 months) during the pandemic (96 women provided free text answers regarding breastfeeding)	Cross‐sectional observational study	Online survey with open questions	Content analysis approach (main themes identified)	The main challenges faced by breastfeeding women were limited access to lactation support, lack of time due to caring responsibility for other children, stress due to the pandemic (affecting milk supply), and financial difficulties. Breastfeeding was viewed as beneficial to transfer antibodies from mother to infant.
Maria et al. (2022) (India)	To examine the facilitators and barriers for breastfeeding and newborn care in government hospitals of Delhi during the initial months of the COVID‐19 pandemic.	Head or senior faculty from Paediatrics/Neonatology (*n* = 12) and Obstetrics (*n* = 7) departments, resident doctors (*n* = 14), nurses (*n* = 13), COVID‐19 negative recently delivered mothers (RDMs) with a healthy newborn (*n* = 18), COVID‐19 infected RDM with a healthy newborn (*n* = 19) or sick newborn (*n* = 8), family members (*n* = 39)	Exploratory descriptive qualitative design	In‐depth interviews (via Google Meet and WhatsApp)	Responses coded thematically using the grounded theory approach	There were mixed experiences; while few mothers resisted breastfeeding due to fear of transmission of infection to the newborn, others preferred breastfeeding to avoid storage of expressed breastmilk, fearing infection risk. Mothers expressed a lack of breastfeeding advice from health care professionals.
Munyan and Kennedy (2022) (United States)	To examine the extent of perceived informational social support within an online virtual community for breastfeeding mothers and identify the virtual community features that breastfeeding mothers found most supportive.	56 members of a social media community of breastfeeding mothers moderated by lactation consultants	Cross‐sectional study	Online survey with open‐ended questions	Content analysis	Participants valued receiving lactation support virtually, especially during COVID‐19 restrictions. Other positives included convenience, the presence of professionals, and accessibility. The lack of in‐person support for breastfeeding issues was a limitation.
Nuampa et al. (2022) (Thailand)	To determine the factors associated with 6‐month exclusive breastfeeding and to explore the experiences of those who exclusively breastfed during the COVID‐19 pandemic.	390 postpartum women; 15 mothers who breastfed exclusively for 6 months participated in qualitative interviews	Mixed methods study (explanatory sequential approach)	In‐depth semi‐structured interviews via video call	Content analysis	Five themes: “(1) a protective shield, (2) I have to save money, (3) I could spend all my time with my baby and breastfeed, (4) spousal support is valuable and (5) opportunity to avoid the obstructed beliefs about exclusive breastfeeding” (p.1)
Okinarum and Rochdiat (2022) (Indonesia)	To investigate mothers' breastfeeding experiences during the COVID‐19 pandemic, including the factors that positively and negatively impacted their breastfeeding experience.	Nine breastfeeding mothers (< 3months postpartum) with mild to moderate stress	Exploratory qualitative design	Face‐to‐face interviews using open‐ended questions	Thematic analysis	Themes for strengthening elements: “(1) maternal affection to her baby, (2) support system from family and community, (3) having adaptive coping strategy” (p.1) Themes for weakening factors: “(1) impaired comfort, (2) insufficient milk supply, (3) financial problem, (4) parenting problem, (5) indifferent husband” (p.1)
Oluoch‐Aridi et al., 2020 (Kenya) YSC start here	To examine the effect of the COVID‐19 pandemic and its preventive strategies on women's experiences of receiving maternity care.	71 postpartum women	Qualitative study	Semi‐structured interviews via telephone	Thematic analysis	Participants experienced stress due to a lack of adequate breast milk supply to feed the baby, which they attributed to the lockdown.
Ombere et al. (2022) (Kenya)	To understand the effect of the COVID‐19 pandemic on maternal health choices, including birth plans.	15 pregnant or postpartum women	Qualitative study	In‐depth interviews and informal interviews	Content analysis	Women supplemented breastfeeding with other available foods since the breast milk was insufficient to meet the baby's nutritional needs due to the mother's inadequate food intake.
Palmquist et al. (2022) (United States)	To examine the intentions, practices and experiences of breastfeeding during the COVID‐19 outbreak.	1437 individuals who fed an infant below 2 years of age during the pandemic	Cross‐sectional mixed methods study	Online survey with open‐ended questions	Content analysis	Three main themes: '(1) emerging knowledge and perceptions of the relationship between COVID‐19 and breastfeeding, human milk, (2) perceptions of immune factors in human milk and COVID‐19, (3) social construction of COVID‐19 and infant and young child feeding perceptions and knowledge' (p.8) Many respondents sustained breastfeeding or delayed the weaning process because they believed that human milk provided their baby with immunological benefits.
Panda et al. (2021) (Ireland)	To explore the perspectives and experiences of women regarding maternity care during the initial COVID‐19 lockdown in Ireland.	19 women who delivered in April–May 20	Qualitative descriptive study	Semi‐structured interviews via telephone	Thematic analysis	Many women experienced difficulty accessing breastfeeding support and advice, with some perceiving telehealth consultations as unhelpful. During the establishment of breastfeeding, women valued privacy due to visitor restrictions.
Ramadan et al. (2022) (Lebanon)	To determine the barriers and facilitators to breastfeeding and to examine how social media resources can help communities become more resilient to the effects of pandemics and disasters.	Facebook breastfeeding support group members (consisting of breastfeeding mothers, mothers who have breastfed, lactation consultants, nurses and psychologists)	Qualitative study	Breastfeeding‐related posts and comments retrieved	Content analysis (1415 posts and comments analysed)	The ability to breastfeed in the event of a COVID‐19 diagnosis was a recurrent concern expressed in several posts by lactating mothers. Mothers appeared concerned that they could infect their infants.
Rice and Williams (2021) (Canada)	To investigate the effect of the COVID‐19 pandemic policies aimed at reducing infection transmission on the postpartum experiences of women.	57 postpartum women	Qualitative descriptive study	Semi‐structured telephone interviews	Thematic analysis	The inability to access professional and social support affected some women's ability to breastfeed successfully. Online breastfeeding support was perceived as not useful.
Riley et al. (2021) (United Kingdom)	To explore women's pregnancy and postpartum experiences amid the COVID‐19 restrictions in England.	25 women (five pregnant and 20 postpartum)	Qualitative study	Semi‐structured interviews by telephone (*n* = 17) and email (*n* = 8)	Inductive reflexive thematic analysis	Participants experienced difficulty obtaining breastfeeding support, leading some to stop breastfeeding. For some participants, the isolation facilitated their breastfeeding journey. Participants felt that the virtual clinics were not as helpful as face‐to‐face visits for breastfeeding advice and help.
Rodríguez‐Gallego et al. (2022) (Spain)	To investigate the effect of the COVID‐19 pandemic and its preventive measures on the initiation and continuation of breastfeeding.	30 postpartum women with breastfeeding experience during the pandemic	Qualitative descriptive study	In‐depth semi‐structured interviews via phone calls	Reflexive inductive thematic analysis	Five main themes: '(1) information received, (2) unequal support from the professionals during the pandemic, (3) social and family support on breastfeeding, (4) impact of confinement and the social restriction measures, and (5) emotional effects of the pandemic' (p.6)
Sayed et al. (2022) (South Africa)	To investigate COVID‐19 pandemic's effect on breastfeeding practices and to study the association of breastfeeding with maternal depressive symptoms, clinic attendance and hunger.	Pregnant women and mothers (with infants <12 months) using the MomConnect mhealth platform (*n* = 3140 for the first survey and 2287 for the follow‐up survey)	Cross‐sectional study	Mobile short message service (SMS) survey (follow‐up survey with an open‐ended question)	Not stated	Breastfeeding mothers were concerned about their nutrition and milk production due to the lack of food in the house during the pandemic.
Shuman et al. (2022) (United States)	To explore the postpartum experiences of women who delivered babies during the initial 6 months of the COVID‐19 pandemic.	371 postpartum women	Cross‐sectional descriptive design	Web‐based survey	Constant comparative method	Many women had negative breastfeeding experiences due to reduced access to breastfeeding support (e.g., lactation consultants). The pandemic's stress adversely affected some women's breast milk supply.
Silverio et al. (2021) (United Kingdom)	To explore the experiences of women regarding the reorganisation of maternity services during the initial wave of the COVID‐19 pandemic in South London.	23 women who delivered between March and August 20 (and received part of their maternity care before the pandemic)	Qualitative study	Semi‐structured interviews using video conferencing software	Template analysis	Participants expressed dissatisfaction with receiving breastfeeding support virtually and preferred in‐person support to check position and latch.
Sinha et al. (2022) (India)	To assess the usage of health care services related to maternal and perinatal care following the implementation of the COVID‐19 pandemic lockdown in comparison to the period preceding it and to gain insights into the experiences, barriers, and determinants contributing to the limited utilisation of health care services during the lockdown.	199 postpartum women (103 and 96 women who delivered before and after the lockdown, respectively) In‐depth interviews with 25 of 96 women who delivered after the lockdown	Mix of quantitative (community survey) and qualitative methodology	In‐depth interviews	Framework analysis	During the COVID‐19 pandemic, the inability to consume adequate food affected the quantity of breast milk produced by women.
Siwik et al. (2022) (Canada)	To investigate the experiences of postpartum breastfeeding women in a vulnerable situation regarding obtaining support for breastfeeding during the COVID‐19 pandemic.	Seven postpartum breastfeeding women from an at‐risk population	Prospective, longitudinal mixed methods study	Semi‐structured interviews via telephone	Open coding followed by axial coding (interpretive description approach)	Most participants faced challenges addressing their breastfeeding concerns since they had limited access to support from health care professionals, family and friends due to the COVID‐19 restrictions. Participants described difficulty using online support systems and preferred in‐person support. Participants felt the pandemic contributed to a sense of connectedness and the development of resilience.
Snyder and Worlton (2021) (United States)	To investigate the influence of the COVID‐19 pandemic on breastfeeding mothers' perceptions of social support.	29 women giving breast milk in any form (i.e., at the breast or through the bottle) to their child during the pandemic	Cross‐sectional phenomenological qualitative study	Semi‐structured telephonic interviews	Immersion and crystallisation	Mothers perceived reduced support networks and increased stress due to the pandemic. They desired more in‐person support from family, friends and lactation professionals. Tele lactation was considered to be less effective, especially for latch issues. Many mothers felt the extended maternity leave positively impacted their breastfeeding journey. Few mothers expressed apprehension regarding the safe expression of breast milk upon their return to the workplace.
Spatz and Froh (2021) (United States)	To explore the delivery and breastfeeding experiences of mothers during the COVID‐19 pandemic.	Three healthy first‐time mothers	Case series	Virtual interviews	Thematic analysis	Telehealth visits for breastfeeding were not considered adequate, especially for latch‐related issues. Mothers preferred in‐person consultation. Being at home due to the pandemic positively influenced breastfeeding.
Sweet, Muller, et al. (2022a) (Australia and Aotearoa New Zealand)	To examine factors affecting post‐natal care experiences, breastfeeding self‐efficacy and study their effect on breastfeeding outcomes during the COVID‐19 pandemic.	1001 mothers in the early (first 6 months) parenting period	Cross‐sectional study	Online survey with open‐text responses	Content analysis	While some women found breastfeeding challenging due to a lack of lactation support, others felt limited visitors and distractions helped better establish breastfeeding.
Sweet, Wilson, et al. (2022) (Australia)	To investigate the experiences of pregnant and postpartum women regarding maternity care received during the initial wave of the COVID‐19 pandemic.	27 women (nine pregnant and 18 postpartum)	Qualitative descriptive study	Semi‐structured in‐depth interviews via telephone or web‐based platform (Zoom)	Thematic analysis	Women believed that lactation consultation should be considered an essential service.
Turner et al. (2023) (Canada and the United Kingdom)	To investigate the influence of the COVID‐19 pandemic on the breastfeeding experiences of first‐time mothers.	Ten first‐time mothers (five each from Canada and the United Kingdom) who breastfed their infants at least once during the pandemic	Qualitative descriptive study	Semi‐structured remote interviews (Microsoft Teams)	Inductive thematic analysis	“Overarching theme: all on mother. Four sub‐themes: '(1) accessing and advocating for health care, (2) social support, (3) becoming a mother in isolation, and (4) breastfeeding baby' (p.1) Mothers experienced difficulties in breastfeeding due to reduced health care and social support. Virtual breastfeeding support was considered not very helpful by many mothers. Some mothers found fewer visitors helpful, as it allowed them to focus more on bonding with their infant and establishing breastfeeding.
Vik et al. (2023) (Norway)	To determine the quality of care and to examine the experiences and perspectives of women on breastfeeding during different phases of the COVID‐19 pandemic.	2922 women who delivered during the pandemic (1021 women provided free text comments, of which 88 were related to breastfeeding)	Mixed‐method cross‐sectional study	Online questionnaire with an open‐ended question	Systematic text condensation	Themes: '(1) understaffed post‐natal wards, (2) early discharge and a lack of professional support, (3) the importance of breastfeeding support from companion of choice and (4) long‐term consequences' (p.5)
von Rieben et al. (2022) (Australia)	To investigate the lived experiences of mothers during the COVID‐19 pandemic and examine the effect of pandemic restrictions on their perceptions of the health system.	Seven postpartum mothers	Qualitative study (inductive and constructivist approach)	Semi‐structured interviews with open‐ended questions via Zoom	Interpretative phenomenological analysis	Mothers preferred in‐person care to telehealth, especially for breastfeeding, since they felt it was an emotional experience and required physical training.
Walsh et al. (2022) (United States)	To explore new parents' paediatric care experiences and determine their support needs during the COVID‐19 pandemic.	30 mothers of infants born in 2020	Qualitative study	Semi‐structured interviews by phone or video calls	Thematic analysis	COVID‐19 restrictions adversely affected mothers' access to lactation support, and telehealth was not seen as an optimal solution for addressing breastfeeding issues related to latch.
Wilson et al. (2022) (Australia)	To investigate the maternity care experiences of women during the COVID‐19 pandemic.	Pregnant (*n* = 2262) or post‐natal women (*n* = 1102) 3202 participants provided free text responses	Cross‐sectional descriptive design	Online survey with fixed‐choice and open‐ended questions	Content analysis	Visitor restrictions during the pandemic were considered beneficial for successful breastfeeding establishment.
Yip et al. (2022) (Hong Kong, China)	To investigate the lived experiences of women not infected with COVID‐19 on breastfeeding their babies amidst the COVID‐19 pandemic.	20 women having the experience of providing breast milk (either directly at the breast or using a bottle to feed expressed milk) to their infants (0‐12 months) at least once during the pandemic	Qualitative research design (descriptive phenomenological approach)	Semi‐structured interviews by telephone or video calls	Colaizzi's seven‐step analytical method for phenomenological data	Two themes: '(1) positive influences on breastfeeding support during COVID‐19, (2) negative influences on breastfeeding support during COVID‐19' (p.1) Positive influences on breastfeeding included support from spouse, working from home, less visitors, fewer unpleasant opinions, enhanced privacy, and more time to focus on breastfeeding. Negative influences included reduced breastfeeding support, primarily hands‐on breastfeeding coaching, increased caring responsibilities at home, and intense focus on breastfeeding.

Abbreviations: COVID‐19, coronavirus disease 2019; IBCLC, International Board‐Certified Lactation Consultant; n, number of participants; RDM, recently delivered mother.

The methodological quality of the included papers showed that most of these papers lacked information about the philosophical perspective. Several papers did not provide details about the researcher's background and their potential influence on the research process (Supplementary material 2). For each sythesised finding, the confidence in the level of evidence was assessed as ‘moderate’ using the JBI ConQual process. The initial ranking for each paper included was ‘high’. Then, using five of the JBI critical appraisal checklist scores, papers were assessed on ‘dependability’. Most studies scored two to three ‘yes’ responses in each of the sythesised findings, resulting in scores dropping from high to moderate in all six synthesised findings. Finally, the papers were assessed on credibility, and the score for each of the synthesised findings remained unchanged (See Table 1).

### 3.2 Synthesised findings

Five synthesised findings for review question 1, and one synthesised finding for review question 2 were generated (see Table 3).

**Table 3:** Synthesised findings and categories for each review question.

Synthesised finding	Categories
Review question 1: What are women's views and experiences of breastfeeding their infants, including breastfeeding support, during the COVID‐19 pandemic?	
Women's awareness and commitment to breastfeeding during the pandemic	Positive views on the health benefits of breastfeeding
	Divergent opinions on breastfeeding during COVID‐19 infection
	Perceived concerns of virus exposure and food scarcity
	Sources of information
The multifaceted breastfeeding experiences of women during the pandemic	No impact of pandemic on breastfeeding
	Positive impact of social isolation on breastfeeding
	Negative impact of pandemic on breastfeeding
Breastfeeding practices and challenges for working women	Working from home facilitated breastfeeding
	Challenges in balancing work and breastfeeding
Professional support during the pandemic: Navigating breastfeeding in an evolving health care context	Access to support and care seeking
	Experiences and perceptions of support, including remote/telehealth support
	Impact on infant feeding decision and practice
Family and peer support groups during the challenging times of the pandemic	Accessibility of in‐person family and peer support
	Significance of family support especially partner support
	Benefits of online breastfeeding support groups
Review question 2: What were the breastfeeding support needs of women during the COVID‐19 pandemic?	
Diverse breastfeeding support needs amidst the pandemic	Need for informational support
	Need for professional support
	Need for family/peer support
	Need for financial and nutritional support
	Need for psychological/emotional support

#### 3.2.1 Review question 1: What are women's views and experiences of breastfeeding their infants, including breastfeeding support, during the COVID‐19 pandemic?

a) **Synthesised finding 1.1: Women's awareness and commitment to breastfeeding during the pandemic**

Women held positive views on the health benefits of breastfeeding, recognising its immunological importance. They accessed information on breastfeeding from diverse sources. Despite having varied opinions about safety and continuation of breastfeeding in the context of COVID‐19 infection, most women placed a high priority on breastfeeding for the health of their infants and remained committed to it despite the challenges.

(i). ***Positive views on the health benefits of breastfeeding***
  Women recognised breastfeeding as the optimal nutrition for infants, providing antibodies and immunity to fight infections. They felt it should be encouraged, particularly during the unsafe and uncertain times of the pandemic. For example, one woman stated:
*Breastfeeding is very important especially when there is a danger to health ‐ the transfer of antibodies from the mother to the baby.*
  (Magnazi et al., 2022, p.5)
(ii). ***Divergent opinions on breastfeeding during COVID‐19 infection***
  Women had varying viewpoints concerning breastfeeding in the context of a potential COVID‐19 infection. Some women considered it safe to continue breastfeeding with hygiene precautions if infected. They believed breastfeeding when infected with COVID‐19 was even more critical, as breast milk may contain antibodies that can help fight the infection:
*It was kind of so that if you do get COVID it's even more important to feed your baby because, sort of, antibodies and keep your baby healthy*….
  (Jackson et al., 2021, p.8)
  However, other women believed that in the event of infection, either pumping breast milk or ceasing breastfeeding altogether would be more appropriate. The varied views could be due to variations in international and country recommendations, as is evident from a woman's quote:
*That the CDC recommends not breastfeeding and separating mother and child if mom tests positive but the WHO says to continue breastfeeding.*
  (Palmquist et al., 2022, p.8)
(iii). ***Perceived concerns of virus exposure and food scarcity***
  Some women expressed concerns about the risk of infecting their infants during breastfeeding. As one woman stated:
*My main fear is for me to be asymptomatic and pass it on to him.*
  (Rodríguez‐Gallego et al., 2022, p.9)
  Additionally, many women were also concerned about the potential consequences of COVID‐19 infection on their ability to continue breastfeeding. For example, one woman shared her concern:
*…I thought that I had corona because suddenly I had fever and pain, so I was worried how the baby would breastfeed*.
  (Badr & Alghamdi, 2022, p.5)
  In lower and middle‐income countries such as India, South Africa and Kenya, the pandemic‐induced financial constraints and food insecurity heightened concerns among some women regarding their ability to access enough food to sustain breastfeeding and produce an adequate supply of breast milk. One woman described her feelings:
*For our child there are lots of expenses, which are difficult to bear after both my husband's and mother's jobs were lost. My breast milk is also not adequate because I am not able to have enough food.*
  (Sinha et al., 2022, p.7)
(iv). ***Sources of information***

Information from diverse sources influenced women's awareness and dedication to breastfeeding during the pandemic, enabling informed decisions for their infants amidst unprecedented challenges. Despite challenges of inaccurate information, women turned to various sources such as midwives, social media, search engines and television to access breastfeeding‐related information throughout the pandemic.

*The information our midwife gave us was that it was important to breastfeed for the antibodies and that it helped for the baby not to get infected by COVID.*

(Rodríguez‐Gallego et al., 2022, p.7)

*There was a lot of disinformation, I searched the Internet…but as everything was so recent, there was not much.*

(Rodríguez‐Gallego et al., 2022, p.7)

b) **Synthesised finding 1.2: The multifaceted breastfeeding experiences of women during the pandemic**

The breastfeeding experiences of women during the pandemic were diverse, encompassing unchanged or positive and negative experiences. Besides pandemic‐related difficulties, some challenges were inherent in a breastfeeding journey.

(i). ***No impact of pandemic on breastfeeding***
  The pandemic did not affect the breastfeeding experiences of some women. This category included those who had already established breastfeeding for their babies and did not require additional support. One woman stated, '*Nothing has changed'* (Badr & Alghamdi, 2022, p.5).
(ii). ***Positive impact of social isolation on breastfeeding***
  Some women viewed the pandemic‐induced confinement and reduced social interactions as a hidden blessing for their breastfeeding journey.
  The decrease in visitors and outdoor commitments allowed more time for focusing on breastfeeding, particularly beneficial for addressing challenges such as latching difficulties. With fewer distractions and ample time during the initial breastfeeding stages, women could persist and overcome obstacles. This advantage was exemplified by a woman's account:
*I've found breastfeeding quite difficult due to problems with latching, nipple tears/bleeding and lots of pain. Being able to stay home and concentrate on getting the feeding right is the only reason I persisted. If I'd had lots of visitors or pressure to meet other mums/family/friends, I'm not sure I would have managed as I've only just got the hang of feeding 5 weeks in!*
  (Brown & Shenker, 2021, p.8)
  The reduction in distractions provided a more peaceful environment, enabling better understanding of the baby's needs. A woman described her experience:
  … *it was a lot more peaceful, and I feel like I easily understand what [baby's] needs were because we didn't have the interruptions of visitors and it was a bit more predictable in what he needed, like when he needed a feed*.
  (Atchan et al., 2023, p.5)
  Additionally, fewer visitors meant less unsolicited advice, as this woman expressed:
*We just did what worked for us and focused on what worked for us without listening to unsolicited advice that you anyway get from family and friends*.
  (Turner et al., 2023, p.5)
  The physical ‘togetherness’ of mother and baby made breastfeeding easier and relaxed, fostering a stronger bonding experience:
*Like I'm here all the time, I'm not going out anywhere. Like there's really no reason to need the bottle*.
  (Turner et al., 2023, p.6)
(iii). ***Negative impact of pandemic on breastfeeding***

The stress and emotional toll of the pandemic were believed by some women to affect their breast milk supply, leading to difficulties. One woman stated:

*Because of the stress and sadness of this event, my breastmilk supply stopped. This experience [was] because of Covid [and] very difficult for my family and I.*

(Shuman et al., 2022, p.105)

Struggles with breastfeeding due to inadequate support triggered emotions of distress, anxiety and frustration, especially among primiparous women. As a woman explained:

*Because you know, you've not got that support there to say ‘No that's fine you don't need to worry about that.’ People there to remind you that babies are resilient and that kind of thing*.

(Turner et al., 2023, p.5)

For multiparous women, the challenges were further compounded by the need to care for other children at home during lockdown. This resulted in limited time to dedicate to breastfeeding.

*It is hard to breastfeed for a long time, with other children at home, (I have 6‐year‐olds twins) and so when they were at home, I gave more formula than I would have given had they not been at home.*

(Magnazi et al., 2022, p.5)

c) **Synthesised finding 1.3: Breastfeeding practices and challenges for working women**

The pandemic led to diverse breastfeeding experiences among working women, particularly between those who changed to remote work and those in essential services such as health care. Home‐based work provided an opportunity for increased bonding and responsive feeding, while essential workers faced challenges due to limited time and demanding workloads.

(i). ***Working from home facilitated breastfeeding***
  The change to remote working arrangements during the pandemic provided a silver lining for many women, providing them increased time to spend and bond with their infants. This closeness allowed for more responsive feeding and eased worries about the practical complexities of pumping breast milk. For example, one woman said:
*It has allowed for me to breastfeed (without pumping) and spend more time than expected with my infant which has been nice*.
  (DeYoung & Mangum, 2021, p.7)
(ii). ***Challenges in balancing work and breastfeeding***
  Women employed in health care settings encountered unique challenges when it came to balancing their work responsibilities with breastfeeding, setting them apart from those who worked from home. The demanding workload, extended shifts, the use of personal protective equipment and limited time for lactation breaks during the pandemic affected their ability to sustain breastfeeding. The high levels of stress and reduced time for expressing breast milk negatively impacted their milk supply. A nurse shared her experience:
*I'm a nurse and so it made work too busy for me to get to pump as often as I wanted. My milk dried up faster and I had to stop early*.
  (DeYoreo et al., 2023, p.157)

Furthermore, concerns about potential contamination and the risk of transmitting infections made many women wary of pumping breast milk at their workplaces, especially within hospital environments. One woman expressed her concerns, saying,

*…I feared getting infected; hospitals are risky areas. I feel it is unsafe to breast pump anywhere—which clinics to go to? Is it hygienic enough? I was concerned*.

(Jiravisitkul et al., 2022, p.9)

d) **Synthesised finding 1.4: Professional support during the pandemic: Navigating breastfeeding in an evolving health care context**

During the pandemic, several breastfeeding women faced challenges due to disrupted post‐natal care, cancelled breastfeeding classes and insufficient professional support, leading to feelings of isolation and uncertainty. The transition to telehealth assistance also imposed limitations. These factors influenced breastfeeding decisions and feeding approaches for some women.

(i). ***Access to support and care seeking***
  During the pandemic, access to post‐natal care faced disruptions, with in‐home visits by health visitors and breastfeeding classes being cancelled. Illustrating this, a woman expressed her frustration:
*Getting access to services postpartum has been difficult, especially with breastfeeding help. I have struggled mentally because I feel unsupported and isolated*.
  (DeYoung & Mangum, 2021, p.7)
  Furthermore, certain regions even classified lactation consultants as nonessential services, compounding the obstacles faced by some breastfeeding women:
*Breastfeeding is really tough… lactation consultants weren't able to do business which makes it harder.*
  (Sweet, Wilson, et al., 2022, p.226)
  However, there were also instances where the fear of virus transmission and the risk of becoming infected discouraged some women from seeking professional support. A woman expressed her concerns:
*… I didn't try to find any [formalized] support yet, because I was not feeling safe to bring him to any group or anything.*
  (Siwik et al., 2022, p.428)
(ii). ***Experiences and perceptions of support, including remote/telehealth support***
  The strain on health care facilities and shortage of available health care professionals resulted in inadequate support for some breastfeeding women, leaving them feeling neglected and unsupported during a crucial time. For instance, a woman shared her distressing experience:
*I need support to get her to latch at every feed and I didn't kind of have that because they were so over stretched. Because of COVID all the rooms had been clamped down*.
  (Turner et al., 2023, p.4)
  Some women reported limited information on breastfeeding during their hospital stay, hindering successful establishment of breastfeeding:
*There's really very little information on breastfeeding in the hospital, nobody who comes and explains anything, or even attentive of what happens to the mother and the baby.*
  (Rodríguez‐Gallego et al., 2022, p.7)
  Lack or inconsistent guidance on breastfeeding while inflected with COVID‐19 or taking medications led to confusion for some women. A lactating woman stated:
*…when I had COVID‐19, I was advised to separate from my child, but then I had also decided on my own that I needed to because—there was no information that was given to me that I could continue safely breastfeeding my baby while I had COVID‐19. So, we were separated, and I didn't breastfeed her after I got COVID‐19*.
  (Ahoya et al., 2023, p.11)
  A woman who experienced this dilemma stated:
*The physicians are divided, some say you can breastfeed the baby while taking medication. I talked to two different physicians at the hospital and they said very different things*
  (Asci et al., 2022, p.7)
  Despite challenges, some women acknowledged the valuable support provided by midwives in both hospital and primary care settings. They considered this support essential for gaining knowledge about breastfeeding techniques and successfully establishing breastfeeding. One woman shared her positive experience,
*I do feel like my midwives were very supportive in the beginning and having them I think was really [unique]*.
  (Turner et al., 2023, p.5)
  Amidst the COVID‐19 restrictions, telehealth appointments, involving phone and video calls, emerged as the primary means of communicating with health care professionals. However, many women found these insufficient and encountered difficulties in conveying and receiving appropriate support for breastfeeding challenges through virtual platforms. One woman shared her experience, expressing her desperation to make breastfeeding work:
*I had a couple of virtual appointments [with lactation consultants], which I found totally useless. I was so desperate to make it work because I had such a wonderful experience nursing my first kid…*.
  (Rice & Williams, 2021, p.E561)
  Women struggled to demonstrate their breastfeeding techniques while holding a baby, a breast and a phone simultaneously:
  '*It's very difficult to show people what I'm doing when I'm trying to maneuver a baby, a boob and a phone'*.
  (Turner et al., 2023, p.5)
  However, some women felt video visits allowed breastfeeding from one's usual environment. As one woman stated:
*Just getting out of the house with an infant is pretty hard, so being able to be in your own home was… also nice because I was sitting in the chair that I breastfeed him in a lot, so it was like… you know, more in my own environment than in an office.*
  (Glassman et al., 2022, p.423)
(iii). ***Impact on infant feeding decision and practice***
  Insufficient professional support had an impact on the infant feeding decisions of some women, leading them to resort to alternative feeding methods. Consequently, some women tended to express breast milk, introduce formula, or engage in mixed feeding. For instance, the absence of access to a lactation consultant due to the pandemic compelled a woman to exclusively pump:

*I was unable to successfully breastfeed and couldn't see a lactation consultant because of the virus. Therefore, breastfeeding was unsuccessful, and I have to exclusively pump*.

(Goyal et al., 2022, p.82)

e) **Synthesised finding 1.5: Family and peer support groups during the challenging times of the pandemic**

Amidst the pandemic, breastfeeding women received limited face‐to‐face assistance from family and friends, with husbands/partners stepping into pivotal roles. In the absence of in‐person peer support, online platforms such as Facebook emerged as valuable resources for obtaining breastfeeding guidance.

(i). ***Accessibility of in‐person family and peer support***
  The pandemic's restrictive measures limited the availability of in‐person social support from family and friends, leaving some women without the emotional and practical assistance crucial for their breastfeeding journey. One woman expressed the impact, stating:
*If a family member had been allowed to visit or something like that just to give me that emotional support. I think that would have helped*.
  (Turner et al., 2023, p.4)
(ii). ***Significance of family support, especially partner support***
  Given restricted access to extended family, husbands/partners emerged as crucial sources of assistance and encouragement for breastfeeding women. Some husbands, who were furloughed or working from home, played an integral role in caring for their baby. They provided practical support with household chores and supported breastfeeding. One woman stated:
*My partner has been furloughed so he is here everyday with us, he can help with nappy changes, looking after our baby and letting me sleep when I need to, basically everything I'm addition to enjoying so many special moments together seeing our baby develop, having 2 of us here all the time means there's much more time for me to focus on breastfeeding our baby*.
  (Brown & Shenker, 2021, p.9)
  The encouragement provided by husbands and partners motivated some women to persist in breastfeeding. For example:
*My partner has been the main support, I breastfed, but he cheered me.*
  (Rodríguez‐Gallego et al., 2022, p.8)
  However, unsupportive husbands posed barriers to breastfeeding, as some urged formula feeding when the baby cried:
*My husband asks me to give formula feeding whenever the baby cries*.
  (Badr & Alghamdi, 2022, p.6)
  Beyond husbands/partners, the support from immediate family members, especially the grandmothers of the baby, was a crucial element in providing advice and motivation for breastfeeding. Even amidst the constraints of lockdown, these family members provided encouragement and guidance through phone conversations, as described below by a woman:
*No one from my family could come because of the quarantine and they therefore could not be of much support. I only had my husband with me anyway. He supported me regarding breastfeeding. But my mother called all the time and encouraged me to breastfeed, breastfeed, breastfeed*.
  (Rodríguez‐Gallego et al., 2022, p.5)
(iii). ***Benefits of online breastfeeding support groups***

In the absence of professional and family support, some women established peer support networks through online platforms, such as Facebook. These platforms enabled them to openly share their concerns, learn from their peers' experiences and access valuable advice easily. Reflecting on her experience, one woman shared:

*In that time, I felt alone. She (breastfeeding mothers from peer support groups via online platform) felt like someone who is close to me and gives me all experience that I need. It was almost like we were friends. I did not even know her face‐to‐face. However, I really valued what she said; I would call it a friendship*…*.*

(Yip et al., 2022, p.6)

#### 3.2.2 Review question 2: What were the breastfeeding support needs of women during the COVID‐19 pandemic?

(a) ***Synthesised finding 2: Diverse breastfeeding support needs amidst the pandemic***

Breastfeeding women had a range of support needs during the pandemic, including precise and reliable information, easily accessible in‐person lactation assistance, emotional support from family, financial stability, food security and psychological well‐being.

(i). ***Need for informational support***
  The need for clear and consistent information regarding breastfeeding was evident among women. Many found themselves uncertain about the safety of breastfeeding when infected with COVID‐19, primarily due to conflicting advice from organisations and health care professionals. This contradictory advice led to a state of confusion.
  The frustration of a woman echoes the challenge of navigating through mixed messages:
*… The person who brought me the medication told me I could breastfeed 3 h later. I called 184 (the COVID helpline) after my baby became feverish. They said I should not have breastfed. I would have liked to have received more accurate information. Hearing confusing things creates a dilemma for a person*.
  (Asci et al., 2022, p.5)
(ii). ***Need for professional support***
  The need for accessible, in‐person lactation support was evident among women struggling with breastfeeding issues. Many women expressed a strong desire for face‐to‐face consultations. A woman shared her perspective, highlighting the significance of in‐person breastfeeding support:
*I was also referred by my health visitor for a breastfeeding Zoom call. That was ridiculous. I needed to see someone face‐to‐face because they have to check your position, your latch and whether your baby has tongue tie. Feeding support has to be there face‐to‐face and it needs to be available*.
  (Silverio et al., 2021, p.5)
  Furthermore, women highlighted the pivotal role of lactation consultants during the pandemic due to the absence of traditional support networks and felt they should be considered an essential service:
*The lactation consultant…is more important with COVID. Because you don't have your mum or your granny…around you…to…help and correct you, you're on your own.*
  (Panda et al., 2021, p.4)
(iii). ***Need for family/peer support***
  Women expressed a strong desire for support from family and peers, a need that was hindered by pandemic‐related restrictions. The absence of emotional support from family members was particularly felt by many women. In cases where support from extended family members was limited, the support of husbands became especially crucial.
  A mother expressed her sentiments about the absence of her mother's physical presence:
*My mother is wonderful and a huge supporter of breastfeeding. I was really looking forward to her coming to visit after my baby was here. She cannot come and whilst we can video message it's just not the same as having your mum close by. I feel I need her, not just to help but emotionally and I'm struggling without this support.*…
  (Brown & Shenker, 2021, p.10)
  Another woman highlighted the integral role her husband played in filling the support gap:
*My husband has had to step up a lot. Like he's taken days off of work sometimes when it's just, I get overwhelmed and it's just hard. He's taken time off so that I can just lay in bed all day or have a bath*.
  (Turner et al., 2023, p.4)
(iv). ***Need for financial and nutritional support***
  Securing financial stability within households with breastfeeding women is imperative to ensure adequate nutrition. The impact of economic strain and food insecurity is illustrated below:
*But my child became so weak and I had to give him other foods including boiled water because I had no milk in my breast because we lacked food in the house (informal conversation with a mother).*
  (Ombere et al., 2022, p.60)
(v). ***Need for psychological/emotional support***
  The pandemic amplified feelings of isolation, stress and anxiety among some women, primarily driven by fears of viral exposure and limited support systems. These experiences highlight the requirement for psychological support. One woman's experience highlights the heightened stress and anxiety:

*Mainly it was probably a lot more stressful um I don't know if that has to do anything with my supply going down and like work all included in there, I'm not sure how that affected it but it's a lot more stressful I know that.*

(Snyder & Worlton, 2021, p.42)

## 4 DISCUSSION

The review findings showed varying opinions among women regarding breastfeeding in the context of a COVID‐19 infection, despite their recognition of its health benefits. These findings align with quantitative research studies (Abuidhail et al., 2022; Gebretsadik et al., 2022). The divergence in opinions may be attributed to discrepancies in global practices, including the separation of infected mothers and infants due to concerns about COVID‐19 infection (Turner et al., 2022; Vu Hoang et al., 2020), despite the WHO's recommendation of skin‐to‐skin contact and direct breastfeeding for COVID‐19‐positive or suspected women (World Health Organization, 2020). Since separating the mother–infant dyad can have adverse effects and impact breastfeeding practices (Bernstein et al., 2023), it should be discouraged without supporting evidence. Considering that women used diverse sources to access breastfeeding information, it is essential to utilise diverse communication channels for effectively disseminating breastfeeding information (Vilar‐Compte et al., 2021). As financial and food security influenced breastfeeding practices (Nuampa et al., 2022), ensuring families' food security, through government support or direct provisions, becomes vital for maternal and child well‐being (Chien et al., 2022; Santana et al., 2023).

Throughout the pandemic, a wide range of breastfeeding experiences emerged. Quantitative studies have also demonstrated varying effects of the pandemic on breastfeeding (Chertok et al., 2022; Chien et al., 2022; Latorre et al., 2021). Some women found lockdowns as beneficial for breastfeeding, citing increased privacy and bonding opportunities, which are critical factors in the early stages of breastfeeding (Gaboury et al., 2017). The shift to remote working and extended maternity leaves during the pandemic enabled direct breastfeeding, increasing maternal confidence and decreasing pumping fatigue (Innstrand et al., 2022; Snyder & Worlton, 2021; Topothai et al., 2022). For breastfeeding support, workplaces should offer remote work flexibility, sufficient maternity leave, and secure, hygienic spaces for pumping and storing breast milk (Badr & Alghamdi, 2022; Rabinowitz & Rabinowitz, 2021). Many women prioritised breastfeeding during the pandemic due to its immune‐boosting benefits, consistent with quantitative research (Costantini et al., 2021). However, some women faced challenges due to limited support, added caregiving responsibilities, heightened stress, isolation and loneliness, aligning with previous systematic reviews' findings (Antoniou et al., 2023; Pacheco et al., 2021), highlighting the need for mental health support (Coca et al., 2023).

During the pandemic, a lack of professional breastfeeding support was observed, which may be partly due to strained health care systems (Koontalay et al., 2021; Mehta et al., 2021), compounded by health care providers' unfamiliarity with COVID‐19‐related breastfeeding guidelines (Kebede et al., 2021). The shift to remote health care introduced additional challenges, prompting a need for innovative solutions such as e‐resources, care maps and prioritising face‐to‐face consultations for physical challenges while utilising online platforms for informational support (Schindler‐Ruwisch & Phillips, 2021; Turner et al., 2023).

Husbands emerged as pivotal sources of support during the pandemic when the conventional support networks were compromised, in line with literature (Antoniou et al., 2023; Vazquez‐Vazquez et al., 2021). A systematic review has shown that father's support enhances breastfeeding outcomes (Koksal et al., 2022), highlighting the importance in engaging them in breastfeeding sessions/interventions (Chang et al., 2021). Online support groups gained prominence in response to inadequate in‐person professional and peer assistance during the pandemic, offering accessibility, information sharing, reassurance and a sense of community (Coca et al., 2022; Morse & Brown, 2022). While the influence of virtual peer support on breastfeeding outcomes is still evolving (Moon & Woo, 2021), addressing barriers to internet access in marginalised communities is critical to ensure equitable access to high‐quality virtual care (Turner et al., 2023).

The COVID‐19 pandemic is an important public health issue and promoting infant and young child feeding through effective breastfeeding support is essential in all emergency responses (Bilgin & Karabayır, 2024). Continued breastfeeding should be actively supported, complemented by integrated counselling services within health care settings. In future pandemics, it is important to prioritise breastfeeding support and designate lactation consultants as essential services to ensure uninterrupted support. In addition, implementing policies such as protected post‐natal time, extended maternity leaves and supportive work arrangements, particularly in countries with limited leave policies, is vital. Policies to support workplace breastfeeding should be in place to ensure employees are able to have sufficient lactation breaks and there are appropriate facilities for breastfeeding in workplaces, particularly in demanding sectors such as health care.

The COVID‐19 pandemic has highlighted the evolving roles of fathers and other support persons, underscoring the importance of tailored breastfeeding support for the families. Clear public health messaging is essential to dispel myths, such as misconceptions about breast milk adequacy based on maternal food intake. There is a need to establish robust mechanisms for food security during crises which can further enhance emergency response efforts to ensure the nutritional needs of vulnerable populations are met effectively. These measures will collectively strengthen emergency preparedness and response strategies, safeguarding the well‐being of infants and young children in times of a pandemic.

### 4.1 Strengths and limitations

A robust and comprehensive search was conducted, without imposing restrictions based on the country of origin. JBI ConQual was rigorously conducted to conclude that the level of confidence of all the synthesised findings was ‘moderate’. However, there are limitations of the review. The inclusion of English‐language publications only could potentially introduce bias. Most of the included studies in this review originated from high and upper‐middle‐income countries. Therefore, the transferability of the findings to broader settings may be restricted.

## 5 CONCLUSION

The review highlights the wide spectrum of breastfeeding experiences that women had during the pandemic. While the lack of social support for those facing breastfeeding difficulties stood out, some women had positive experiences due to the lockdown‐induced social isolation. Despite the shift of numerous appointments to virtual platforms, the review highlights the need for clear and consistent information, psychological/emotional support, accessibility to lactation services. Food security should be provided for economically underprivileged breastfeeding women during times of crisis.

## AUTHOR CONTRIBUTIONS

Yan‐Shing Chang initiated and conceptualised the review. The review questions and design were developed by Padma Uma Devi and Yan‐Shing Chang. Padma Uma Devi conducted the literature search, with Yan‐Shing Chang's guidance. Padma Uma Devi, Yan‐Shing Chang and Sarah Beake screened the full texts. Padma Uma Devi carried out data extraction and data analysis, with support of Yan‐Shing Chang and Sarah Beake. Padma Uma Devi, Yan‐Shing Chang, and Sarah Beake undertook quality assessment. Padma Uma Devi prepared the initial draft with critical input of Yan‐Shing Chang and Sarah Beake. All authors read and approved the final version of the manuscript.

## CONFLICT OF INTEREST STATEMENT

The authors declare no conflicts of interest.

## Supporting information

Supporting information.

Supporting information.

## Data Availability

The data that support the findings of this study are available from the corresponding author upon reasonable request.
