# Supplementary material for: Women's views and experiences of breastfeeding during the coronavirus disease 2019 pandemic: A systematic review of qualitative evidence
Source: Matern Child Nutr. 2024 Aug 9;20(4):e13708. doi: 10.1111/mcn.13708 (PMC11574658; doi:10.1111/mcn.13708)
Supplement: Supplementary file 1 — Supporting information. [file MCN-20-e13708-s001.docx]

**Supplementary material 1**

**SEARCH STRATEGY USED ON THE VARIOUS DATABASES**

1. **Full search strategy on MEDLINE (OVID)**

|  | **Searches** | **Results** |
| --- | --- | --- |
| 1 | exp breast feeding/ | 43441 |
| 2 | (breastfeed* or breastfed or breast feed* or breast fed or breast-feed* or breast-fed).mp. | 67019 |
| 3 | exp lactation/ | 47448 |
| 4 | (lactation or lactating).mp. | 76079 |
| 5 | (infant feed* or infant-feed* or infant* fed).mp. | 8810 |
| 6 | bottle feeding/ | 3985 |
| 7 | (bottle feed* or bottle fed or bottle-feed* or bottle-fed or bottlefeed* or bottlefed).mp. | 5820 |
| 8 | (finger feed* or finger-feed* or fingerfeed* or finger fed or finger-fed or fingerfed).mp. | 28 |
| 9 | (cup feed* or cup-feed* or cupfeed* or cup fed or cup-fed or cupfed).mp. | 73 |
| 10 | milk, human/ | 22332 |
| 11 | (human milk or breast milk or breastmilk or breast-milk).mp. | 28417 |
| 12 | colostrum.mp. or colostrum/ | 10337 |
| 13 | 1 or 2 or 3 or 4 or 5 or 6 or 7 or 8 or 9 or 10 or 11 or 12 | 157490 |
| 14 | limit 13 to (yr="2020 -Current" and covid-19) | 1409 |

1. **Full search strategy on Embase (1974) (OVID)**

|  | **Searches** | **Results** |
| --- | --- | --- |
| 1 | exp breast feeding/ | 65029 |
| 2 | (breastfeed* or breastfed or breast feed* or breast fed or breast-feed* or breast-fed).mp. | 85935 |
| 3 | lactation/ | 58173 |
| 4 | (lactation or lactating).mp. | 81187 |
| 5 | exp infant feeding/ | 72512 |
| 6 | (infant feed* or infant-feed* or infant* fed).mp. | 15617 |
| 7 | bottle feeding/ | 4365 |
| 8 | (bottle feed* or bottle fed or bottle-feed* or bottle-fed or bottlefeed* or bottlefed).mp. | 5880 |
| 9 | (finger feed* or finger-feed* or fingerfeed* or finger fed or finger-fed or fingerfed).mp. | 40 |
| 10 | (cup feed* or cup-feed* or cupfeed* or cup fed or cup-fed or cupfed).mp. | 80 |
| 11 | exp breast milk/ | 33527 |
| 12 | (breast milk or breastmilk or breast-milk or human milk).mp. | 44179 |
| 13 | colostrum.mp. or exp colostrum/ | 10932 |
| 14 | 1 or 2 or 3 or 4 or 5 or 6 or 7 or 8 or 9 or 10 or 11 or 12 or 13 | 188067 |
| 15 | limit 14 to (yr="2020 -Current" and covid-19) | 2397 |

1. **Full search strategy on CINAHL (EBSCO)**

|  | **Searches** | **Results** |
| --- | --- | --- |
| S1 | MH "Breast Feeding+" | 27,195 |
| S2 | breastfeed* or breastfed or “breast feed*” or “breast fed” or breast- feed* or breast-fed | 37,025 |
| S3 | MH Lactation | 4,891 |
| S4 | lactation or lactating | 9,860 |
| S5 | MH "Infant Feeding+" | 31,003 |
| S6 | “infant feed*” or infant- feed* or “infant* fed” | 8,323 |
| S7 | MH "Bottle Feeding" | 1,922 |
| S8 | “bottle feed*” or “bottle fed” or bottle-feed* or bottle-fed or bottlefeed* or bottlefed | 2,421 |
| S9 | “finger feed*” or finger- feed* or fingerfeed* or “finger fed” or finger-fed or fingerfed | 15 |
| S10 | “cup feed*” or cup-feed* or cupfeed* or “cup fed” or cup-fed or cupfed | 58 |
| S11 | MH "Milk, Human+" | 7,657 |
| S12 | “human milk” or “breast milk” or breastmilk or breast-milk | 9,864 |
| S13 | MH Colostrum or Colostrum | 1,016 |
| S14 | S1 OR S2 OR S3 OR S4 OR S5 OR S6 OR S7 OR S8 OR S9 OR S10 OR S11 OR S12 OR S13 | 50,144 |
| S15 | (((MH "Coronavirus+") or (MH "Coronavirus Infections+") or (coronavirus* or corona virus* or OC43 or NL63 or 229E or HKU1 or HCoV* or ncov* or covid* or sars-cov* or sarscov* or Sars-coronavirus* or Severe Acute Respiratory Syndrome Coronavirus*)) not ((SARS or SARS- CoV or MERS or MERS- CoV or Middle East respiratory syndrome or camel* or dromedar* or equine or coronary or coronal or covidence* or covidien or influenza virus or HIV or bovine or calves or TGEV or feline or porcine or BCoV or PED or PEDV or PDCoV or FIPV or FCoV or SADS-CoV or canine or CCov or zoonotic or avian influenza or H1N1 or H5N1 or H5N6 or IBV or murine corona*)) or (MH "COVID-19+") or (MH "COVID-19 Pandemic") or (MH "SARS-CoV-2") or (covid or 2019-ncov or ncov19 or ncov-19 or 2019-novel CoV or sars-cov2 or sars- cov-2 or sarscov2 or sarscov-2 or Sars- coronavirus2 or Sars- coronavirus-2 or SARS- like coronavirus* or coronavirus-19 or ((novel or new or nouveau) N2 (CoV or nCoV or coronavirus* or “corona virus” or Pandemi*)) or ((subvariant* or variant*) adj2 (India* or "South Africa*" or UK or English or Brazil* or alpha or beta or delta or gamma or kappa or lambda or mu or "AY.X" or "BA.1" or "BA.2" or "BA.3" or "BA.4" or "BA.5" or "P.1" or "C.37")) or ("B.1.1.7" or "B.1.351" or "B.1.617.1" or "B.1.617.2" or "B.1.1.529*" or "B.1.61.7*" or "21L/BA.2" or "21K/BA.1" or “XBB.1.5”) or Deltacron or Omnicron) and EM 20190601- 20301231 | 130,711 |
| S16 | S14 AND S15 | 834 |

1. **Full search strategy on Web of Science (Core Collection)**

|  | **Searches** | **Results** |
| --- | --- | --- |
| 1 | TS=(breastfeed* or breastfed or “breast feed*” or “breast fed” or breast-feed* or breast-fed or lactation or lactating or “infant feed*” or infant-feed* or “infant* fed” or “bottle feed*” or “bottle fed” or bottle-feed* or bottle-fed or bottlefeed* or bottlefed or “finger feed*” or finger-feed* or fingerfeed* or “finger fed” or finger-fed or fingerfed or “cup feed*” or cup-feed* or cupfeed* or “cup fed” or cup-fed or cupfed or “human milk” or “breast milk” or breastmilk or breast-milk or colostrum) | 296499 |
| 2 | TS=(coronavirus* or “corona virus*” or OC43 or NL63 or 229E or HKU1 or HCoV* or ncov* or covid* or sars-cov* or sarscov* or Sars-coronavirus* or “Severe Acute Respiratory Syndrome Coronavirus*”) not TS=(SARS or SARS-CoV or MERS or MERS-CoV or “Middle East respiratory syndrome” or camel* or dromedar* or equine or coronary or coronal or covidence* or covidien or “influenza virus” or HIV or bovine or calves or TGEV or feline or porcine or BCoV or PED or PEDV or PDCoV or FIPV or FCoV or SADS-CoV or canine or CCov or zoonotic or “avian influenza” or H1N1 or H5N1 or H5N6 or IBV or “murine corona*”) | 300586 |
| 3 | TS=(covid or 2019-ncov or ncov19 or ncov-19 or “2019-novel CoV” or sars-cov2 or sars-cov-2 or sarscov2 or sarscov-2 or Sars-coronavirus2 or Sars-coronavirus-2 or “SARS-like coronavirus*” or coronavirus-19) | 411101 |
| 4 | TS=((novel or new or nouveau) Near/2 (CoV or nCoV or coronavirus* or “corona virus” or Pandemi*)) | 23661 |
| 5 | TS=((subvariant* or variant*) Near/2 (India* or "South Africa*" or UK or English or Brazil* or alpha or beta or delta or gamma or kappa or lambda or mu or "AY.X" or "BA.1" or "BA.2" or "BA.3" or "BA.4" or "BA.5" or "P.1" or "C.37")) | 15771 |
| 6 | TS=("B.1.1.7" or "B.1.351" or "B.1.617.1" or "B.1.617.2" or "B.1.1.529*" or "B.1.61.7*" or "21L/BA.2" or "21K/BA.1" or “XBB.1.5” or Deltacron or Omnicron) | 2534 |
| 7 | #2 OR #3 OR #4 OR #5 OR #6 | 439570 |
| 8 | (#7) AND DOP=(2019-06-01/2030-12-31) | 425223 |
| 9 | #1 AND #8 | 3035 |
