# Supplementary material for: Women's views and experiences of breastfeeding during the coronavirus disease 2019 pandemic: A systematic review of qualitative evidence
Source: Matern Child Nutr. 2024 Aug 9;20(4):e13708. doi: 10.1111/mcn.13708 (PMC11574658; doi:10.1111/mcn.13708)
Supplement: Supplementary file 2 — Supporting information. [file MCN-20-e13708-s002.docx]

**Supplementary material 2**

**Critical appraisal of included papers as per Joanna Briggs Institute checklist for qualitative research**

| Author(s) and Year | 1. Is there congruity between the stated philosophical perspective and the research methodology? | 2. Is there congruity between the research methodology and the research question or objectives? | 3. Is there congruity between the research methodology and the methods used to collect data? | 4. Is there congruity between the research methodology and the representation and analysis of data? | 5. Is there congruity between the research methodology and the interpretation of results? | 6. Is there a statement locating the researcher culturally or theoretically? | 7. Is the influence of the researcher on the research, and vice- versa, addressed? | 8. Are participants, and their voices, adequately represented? | 9. Is the research ethical according to current criteria or, for recent studies, and is there evidence of ethical approval by an appropriate body? | 10. Do the conclusions drawn in the research report flow from the analysis, or interpretation, of the data? |
| --- | --- | --- | --- | --- | --- | --- | --- | --- | --- | --- |
| Agrina et al. 2022 | Unclear | Yes | Yes | Yes | Yes | Unclear | No | Yes | Yes | Yes |
| Ahoya et al. 2023 | Unclear | Yes | Yes | Yes | Yes | No | No | Yes | Yes | Yes |
| Aşcı et al. 2022 | Unclear | Yes | Yes | Yes | Yes | Unclear | Yes | Yes | Yes | Yes |
| Atchan et al. 2023 | Unclear | Yes | Yes | Yes | Yes | No | Yes | Yes | Yes | Yes |
| Atuhaire et al. 2021 | Unclear | Yes | Yes | Yes | Yes | No | Yes | Yes | Yes | Yes |
| Badr & Alghamdi 2022 | Unclear | Yes | Yes | Yes | Yes | No | No | Yes | Yes | Yes |
| Brown & Shenker 2021 | Unclear | Yes | Yes | Yes | Yes | No | No | Yes | Yes | Yes |
| Cassar & Spiteri 2022 | Yes | Yes | Yes | Yes | Yes | No | No | Yes | Yes | Yes |
| Ceulemans et al. 2021 | Unclear | Yes | Yes | Unclear | Yes | Unclear | No | Yes | Yes | Yes |
| Cohen & Botz 2022 | Yes | Yes | Yes | Yes | Yes | Yes | Unclear | Yes | Yes | Yes |
| DeYoreo et al. 2023 | Unclear | Yes | Yes | Yes | Unclear | No | No | Yes | Yes | Yes |
| DeYoung & Mangum 2021 | Unclear | Yes | Yes | Yes | Yes | No | No | Yes | Yes | Yes |
| Eri et al. 2022 | Unclear | Yes | Yes | Yes | Yes | No | No | Yes | No | Yes |
| Fry et al. 2021 | Unclear | Yes | Yes | Yes | Yes | No | No | Yes | No | Yes |
| Fumagalli et al. 2022 | Yes | Yes | Yes | Yes | Yes | Unclear | Yes | Yes | Yes | Yes |
| Glassman et al. 2022 | Unclear | Yes | Yes | Yes | Yes | No | No | Yes | Yes | yes |
| Goyal et al. 2022 | Unclear | Yes | Yes | Yes | Yes | No | No | Yes | Yes | Yes |
| Hill et al. 2023 | Unclear | Yes | Yes | Yes | Yes | Yes | Yes | Yes | Yes | Yes |
| Jackson et al. 2021 | Unclear | Yes | Yes | Yes | Yes | Yes | Yes | Yes | Yes | Yes |
| Jacob et al. 2022 | Unclear | Yes | Yes | Yes | Yes | No | No | Yes | Yes | Yes |
| Jensen et al. 2022 | Unclear | Yes | Yes | Yes | Yes | No | Yes | Yes | Yes | Yes |
| Jiravisitkul et al. 2022 | Unclear | Yes | Yes | Yes | Yes | No | No | Yes | Yes | Yes |
| Joy et al. 2020 | Yes | Yes | Yes | Yes | Yes | Yes | Yes | Yes | Yes | Yes |
| Kolker et al. 2021 | Yes | Yes | Yes | Yes | Yes | No | No | Yes | Yes | Yes |
| Magnazi et al. 2022 | Unclear | Yes | Yes | Yes | Yes | No | No | Yes | Yes | Yes |
| Maria et al. 2022 | Unclear | Yes | Yes | Yes | Yes | Yes | Unclear | Yes | Yes | Yes |
| Munyan & Kennedy 2022 | Unclear | Yes | Yes | Yes | Yes | No | Unclear | Yes | Yes | Yes |
| Nuampa et al. 2022 | Unclear | Yes | Yes | Yes | Yes | No | No | Yes | Yes | Yes |
| Okinarum & Rochdiat 2022 | Unclear | Yes | Yes | Yes | Yes | No | Unclear | Yes | Yes | Yes |
| Oluoch-Aridi et al. 2020 | Unclear | Yes | Yes | Yes | Yes | Yes | No | Yes | Yes | Yes |
| Ombere et al. 2022 | Unclear | Yes | Yes | Unclear | Unclear | No | No | Yes | Yes | Yes |
| Palmquist et al. 2022 | Unclear | Yes | Yes | Yes | Yes | Unclear | No | Yes | Yes | Yes |
| Panda et al. 2021 | Yes | Yes | Yes | Yes | Yes | Yes | No | Yes | Yes | Yes |
| Ramadan et al. 2022 | Unclear | Yes | Yes | Yes | Yes | No | No | Yes | Yes | Yes |
| Rice & Williams 2021 | Yes | Yes | Yes | Yes | Yes | Yes | Yes | Yes | Yes | Yes |
| Riley et al. 2021 | Unclear | Yes | Yes | Yes | Yes | Yes | Yes | Yes | Yes | Yes |
| Rodriguez-Gallego et al. 2022 | Unclear | Yes | Yes | Yes | Yes | Yes | Yes | Yes | Yes | Yes |
| Sayed et al. 2022 | Unclear | Unclear | Unclear | Unclear | Unclear | No | No | Unclear | Yes | Yes |
| Shuman et al. 2022 | Unclear | Yes | Yes | Unclear | Unclear | No | No | Yes | Yes | Yes |
| Silverio et al. 2021 | Yes | Yes | Yes | Yes | Yes | No | No | Yes | Yes | Yes |
| Sinha et al. 2022 | Unclear | Unclear | Unclear | Unclear | Unclear | No | No | Yes | Yes | Yes |
| Siwik et al. 2022 | Yes | Yes | Yes | Yes | Yes | No | Unclear | Yes | Yes | Yes |
| Snyder & Worlton 2021 | Unclear | Yes | Yes | Unclear | Unclear | No | No | Yes | Yes | Yes |
| Spatz & Froh 2021 | Unclear | Yes | Yes | Yes | Yes | No | No | Yes | Yes | Yes |
| Sweet et al. 2022a | Unclear | Yes | Yes | Yes | Unclear | No | No | Yes | Yes | Yes |
| Sweet et al. 2022b | Unclear | Yes | Yes | Yes | Yes | No | No | Yes | Yes | Yes |
| Turner et al. 2023 | Unclear | Yes | Yes | Yes | Yes | Yes | Unclear | Yes | Yes | Yes |
| Vik et al. 2023 | Unclear | Unclear | Unclear | Unclear | Unclear | No | No | Yes | Yes | Yes |
| VonRieben et al. 2022 | Yes | Yes | Yes | Yes | Yes | Yes | Yes | Yes | Yes | Yes |
| Walsh et al. 2022 | Unclear | Yes | Yes | Yes | Yes | No | Unclear | Yes | Yes | Yes |
| Wilson et al. 2022 | Unclear | Yes | Yes | Yes | Unclear | No | No | Yes | Yes | Yes |
| Yip et al. 2022 | Unclear | Yes | Yes | Yes | Yes | Unclear | Unclear | Yes | Yes | Yes |
